# *Aedes hensilli* as a Potential Vector of Chikungunya and Zika Viruses

**DOI:** 10.1371/journal.pntd.0003188

**Published:** 2014-10-09

**Authors:** Jeremy P. Ledermann, Laurent Guillaumot, Lawrence Yug, Steven C. Saweyog, Mary Tided, Paul Machieng, Moses Pretrick, Maria Marfel, Anne Griggs, Martin Bel, Mark R. Duffy, W. Thane Hancock, Tai Ho-Chen, Ann M. Powers

**Affiliations:** 1 Division of Vector-Borne Diseases, Centers for Disease Control and Prevention, Fort Collins, Colorado, United States of America; 2 URE-Entomologie Medicale, Institut Pasteur de Nouvelle-Caledonie, Noumea, New Caledonia; 3 Environmental Health Services, Division of Public Health, Department of Health Services, Pohnpei, Federated States of Micronesia; 4 National Food Safety Program, Department of Health and Social Affairs, Pohnpei, Federated States of Micronesia; 5 Department of Health, Education and Social Affairs, Pohnpei, Federated States of Micronesia; 6 Wa′ab Community Health Center, Yap, Federated States of Micronesia; 7 Epidemic Intelligence Service Field Assignments Branch, Centers for Disease Control and Prevention, Atlanta, Georgia, United States of America; United States Army Medical Research Institute of Infectious Diseases, United States of America

## Abstract

An epidemic of Zika virus (ZIKV) illness that occurred in July 2007 on Yap Island in the Federated States of Micronesia prompted entomological studies to identify both the primary vector(s) involved in transmission and the ecological parameters contributing to the outbreak. Larval and pupal surveys were performed to identify the major containers serving as oviposition habitat for the likely vector(s). Adult mosquitoes were also collected by backpack aspiration, light trap, and gravid traps at select sites around the capital city. The predominant species found on the island was *Aedes (Stegomyia) hensilli*. No virus isolates were obtained from the adult field material collected, nor did any of the immature mosquitoes that were allowed to emerge to adulthood contain viable virus or nucleic acid. Therefore, laboratory studies of the probable vector, *Ae. hensilli*, were undertaken to determine the likelihood of this species serving as a vector for Zika virus and other arboviruses. Infection rates of up to 86%, 62%, and 20% and dissemination rates of 23%, 80%, and 17% for Zika, chikungunya, and dengue-2 viruses respectively, were found supporting the possibility that this species served as a vector during the Zika outbreak and that it could play a role in transmitting other medically important arboviruses.

## Author Summary

Arthropod-borne viruses (arboviruses) cause significant human morbidity and mortality throughout the world. Zika virus, which is reported to be transmitted by *Aedes* (*Stegomyia*) species mosquitoes, caused an outbreak on the island of Yap, in the Federated States of Micronesia in 2007. This was the first described outbreak of Zika in Oceania, which has had several arbovirus outbreaks in the past. Diagnosing the outbreak was difficult due to the similarity in clinical symptoms between disease caused by Zika virus and other viruses. This work describes the efforts to identify the mosquito species that were responsible for transmission of the virus. While no virus was isolated from any species of mosquito collected during the current study, the predominant species found was *Aedes hensilli* and through the complementary laboratory studies, this mosquito was implicated as a probable vector for Zika virus. In addition, this species was found to be susceptible to both the medically important dengue-2 and chikungunya viruses.

## Introduction

Outbreaks of arboviral disease have been documented in islands of the western Pacific including The Federated States of Micronesia (FSM) and Palau. Multiple dengue outbreaks have been reported in the western pacific [1]–[4] with an outbreak of dengue 4 virus occurring in Palau in 1995 after a 7 year absence of dengue on this island [5]. This first outbreak of dengue 4 in the Western Pacific also affected FSM the same year [6]. Additional dengue outbreaks occurred more recently in FSM during 2004 and 2012–13 [7], [8]. In 2007, an outbreak of acute febrile illness characterized by rash, conjunctivitis, fever, and arthralgia was reported on the island of Yap in the Federated States of Micronesia. While dengue was originally suspected, clinicians noted differences from classical dengue fever and collected serum from acutely-ill individuals for diagnosis. Chikungunya virus (CHIKV) was also considered as the clinical presentation was representative of CHIKV infection and an ongoing epidemic of CHIKV was occurring in Southeast Asia. However, Zika virus (ZIKV) nucleic acid was detected in 14% of the samples tested and no evidence of alternate etiologies was identified [9].

Zika virus (ZIKV) is a member of the family *Flaviviridae*. Presence of the virus in human specimens has been demonstrated by virus isolation (samples from Africa and Asia) and antibody presence (Asia) [10]–[15]; however, only a handful of clinical disease cases were described in the literature prior to this 2007 outbreak [16]–[19]. Since the outbreak in Yap, additional ZIKV outbreaks have been documented in Gabon in 2007 and in French Polynesia in 2013 [20], [21]. Mosquito vectors from which virus has been identified include (among others) *Aedes africanus, Aedes luteocephalus, Aedes aegypti*, and *Aedes albopictus* (all belonging to the subgenus *Stegomyia*) [15], [21]–[25]. However, little else is known regarding the natural ecology of the virus.

Because this was the first documentation of the virus in Oceania, understanding the biological transmission of the virus was a public health priority. A team including epidemiologists, clinicians, entomologists, and public health personnel investigated the outbreak with the objectives of characterizing the epidemiology, course of clinical illness, and ecological factors contributing to the epidemic and transmission of the virus. Household surveys were performed to obtain serum specimens, to obtain clinical and epidemiological data, to identify risk factors for infection, and to collect entomological specimens for the purpose of determining the most probable epidemic vector [9]. The entomological studies included both immature (larval and pupal) and adult surveys to determine the species present on the island, contributions of distinct container types in mosquito maintenance, and to perform virus isolation. This report describes the entomologic findings from the field collected material as well as subsequent laboratory studies assessing the vector capacity of the likely outbreak vector.

## Methods

### Description of the entomological investigation sites

**Figure 1:**
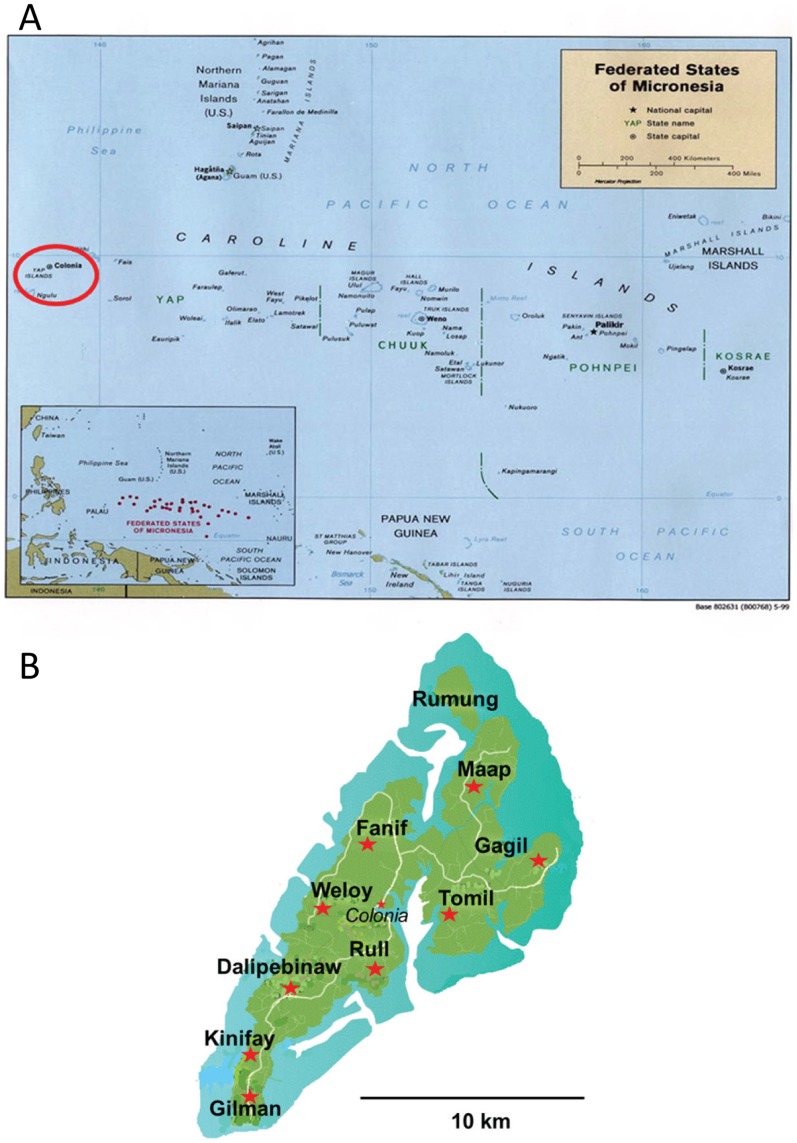
Maps showing size and locations of Yap State (A) and sites of collections on Yap Island (indicated by stars) (B).

The Federated States of Micronesia are located in the Western Pacific Ocean northeast of Papua New Guinea (**Figure 1**). Yap State is the westernmost state and is comprised of a main island group consisting of four closely associated islands situated at 9° North and 138° East. It is approximately 6 km wide by 15 km long with a population of 7,391 persons during the 2000 census. The climate is tropical with warm temperatures and rainfall reported throughout the year. Mosquitoes were collected and household surveys performed between July 4, 2007 and July 16, 2007 at 170 randomly selected homes in 9 out of the 10 municipalities, representing 16% of the total households on the island. The outbreak was estimated to have begun in April, 2007 and continued through July of 2007 [9].

### Adult mosquito collections

Adult mosquito sampling was carried out using three collection methods. Host seeking mosquitoes were collected using light traps, resting mosquitoes were collected using vacuum aspiration, and mosquitoes looking for oviposition habitat were collected with gravid traps. Gravid and light traps (light only) were set in the evening at three sites in the state capital city of Colonia from July 4–9, and July 12–16. Collection bags from the light and gravid traps were recovered daily for 9 days in the early morning. A battery operated backpack or handheld mechanical aspirators were used to collect mosquitoes resting in and around random houses where serosurveys were being performed during daytime hours, July 9–15.

All collected mosquitoes were identified morphologically using keys from Bohart [26], and Rueda [27]. They were then sorted by sex, species, collection method, and collection period and placed into cryovials at a temporary laboratory set up in Colonia. Specimens were frozen at −20°C on-site; they were later transported to the CDC at Fort Collins CO, USA where storage was at −70°C until processing.

### Larval surveys

All indoor and outdoor water containing receptacles at the randomly selected households [9] were inspected for mosquito larvae and pupae. Live larvae observed in receptacles were collected and identified to species and allowed to emerge to confirm identification. All pupae found were collected and reared to adulthood. Key habitat information was recorded and larval indices (Breteau and household [28]) were calculated from the collected data for each of the species observed.

### Mosquito pool virus isolation

Each pool of mosquitoes (not exceeding 40 individuals) were placed into a 1.7 mL polypropylene tube (Eppendorf, Hauppauge, NY) and ground with a pestle (Kontes) and 500 µl of Dulbecco's minimal essential medium (DMEM) (Gibco) supplemented with 10% fetal bovine serum (FBS), 100 U/mL of penicillin and streptomycin, 1 U/mL of fungizone and gentamycin. The homogenized mosquitoes were then centrifuged at 15,000 g for 1 min. Triturate was then transferred to a new tube and frozen at −70°C. 100 µl of thawed triturate was then plated onto a 96-well cell culture plate (Corning). 50 µl of a Vero cell suspension was then added to the same well and placed into an incubator at 37°C and 5% carbon dioxide. The cell and homogenized mosquito mixture was then monitored daily for cytopathic effect (CPE) for 10 days. Medium from those wells presenting signs of CPE (presumptive positives) were removed and placed at −70°C until use [29].

### Virus isolates

The viruses used for laboratory mosquito infections were obtained from the Arbovirus Reference Collection at CDC, Fort Collins, CO (**Table 1**). As no ZIKV field strain was obtained, we used the prototype strain for ZIKV laboratory infections.

**Table 1:** Virus isolates used for laboratory mosquito infections.

Virus	Strain	Origin/Source	Passage History*
Zika	MR 766	Rhesus monkey, Uganda 1947	P149, V2
Dengue 2	Jam 1409	Human, Jamaica 1949	P3, C6(2)
Dengue 2	TR 1751	Human, Trinidad 1953	P55, C6 (1)
Chikungunya	COM 125	Mosquito, Comoros 2005	V2

*P = passage (culture type unspecified), V  =  Vero cells, C6  =  Aedes albopictus C6/36 cells.

### Viral nucleic acid extraction and detection

Viral RNA was isolated using the QiaAmp viral RNA protocol (Qiagen). Total RNA was extracted from 50 µl of cell supernatant (CPE positive pools) or 100 µl of mosquito homogenate (artificial infections) and eluted from the kit columns using 60 µl of elution buffer. The RNA was stored at −70°C until use.

Both reverse-transcription PCR (RT-PCR) and real-time RT-PCR assays were utilized to detect viral nucleic acid. The Titan one-step RT-PCR (Roche) kit was paired with the primers FU1 and cFD3 for detection of Zika [30], [31]. Briefly, 5 µl of sample RNA was added to the kit components and 400 nM of primers. The manufacturer's protocol was followed with no modifications. The reactions were analyzed by gel electrophoresis. The real-time PCR assay was used on both the presumptive positive pools and the experimentally infected mosquitoes. The previously described Zika (800 series set) and chikungunya virus specific primer and probe sets were used [32], [33]. The DENV-2 oligonucleotide set were designed with the Primer Select software program (DNASTAR) (1085 CCAAACAACCCGCCACTCTAAG, 1244c TTTCCCCATCCTCTGTCTACCATA, and TaqMan probe 1145 FAM-AACAGACTCGCGCTGCCCAACACA-BHQ1) and were based on the published GenBank full-length sequences. All real-time assays were performed by using the QuantiTect probe RT-PCR reagent kit (Qiagen). Briefly, a 50µl total reaction volume consisted of kit components, 10 µl of RNA, 400 nM of each primer, and 150 nM of probe. The reactions were subjected to 45 cycles of amplification in an iQ5 Real-Time PCR detection system (BioRad) following the manufacturer's protocol. The limits of detection for DENV, ZIKV, and CHIKV assays were found using the previously described techniques [34] and were cycle threshold (Ct) values of 37.7, 36.1, and 38.0 respectively, which is equivalent to approximately 1.0 plaque forming unit/mL. In addition, each run included a standard RNA curve. The standard curve was completed by serially diluting the virus stock and extracting the RNA from each dilution, according to the previously mentioned RNA extraction protocol, while simultaneously titrating each dilution in a standard plaque assay. A curve correlation coefficient of ≥0.950 and a 90–100% PCR efficiency was used to validate each detection assay.

### Mosquito colonization

Mosquito eggs were collected at selected houses in Yap using oviposition cups. Briefly, black, plastic cups were lined with seed germination paper [35] and filled approximately half full with water. Cups were placed under foliage near selected homes (2–4 feet above the ground) and collected after 3–5 days. Field collected egg liners were wrapped in moist paper towels, sealed in Ziploc-style bags, and transported to the insectary at the Center for Disease Control and Prevention (CDC), Fort Collins Colorado for colonization. The eggs were washed with a 10% bleach solution prior to hatching in a pan of tap water to eliminate surface fungal and bacterial contaminants.

Larvae were supplied with either a liver powder solution or mouse pellets as appropriate for the developmental stage and identified to species as 4^th^ instar. All larvae collected were identified as *Aedes (Stegomyia) hensilli*. Pupation occurred between days 5–7 post hatching. Pupae were removed from the larval pans and allowed to emerge into 1 ft^3^ adult mosquito cage (BioQuip). In order to produce the next generation, adults were provided an anesthetized mouse as a blood meal source and the engorged females were provided with an oviposition site (seed germination paper) to deposit their eggs. The process was repeated in order to get sufficient numbers of experimental mosquitoes. In addition, species verification was performed on F2 adult mosquitoes.

### Laboratory mosquito infections

Three to four day-old adult *Ae. hensilli* mosquitoes (F12–15) were fed on blood meals containing ZIKV, CHIKV, or DENV-2. The blood meals contained equal parts of virus, FBS with 10% sucrose, and sheep blood (Colorado Serum CO) washed with phosphate-buffered saline and packed by centrifugation. A Hemotek feeding system (Discovery Workshops) was used to deliver the blood meal to the mosquitoes for 1 hour at 37°C. The fully engorged females were separated and placed into a humidified environmental chamber (Thermo Scientific) and held at 28°C for 8 days until processing. Blood meal titer was determined by plaque assay to determine input titer.

After the 8 day holding period, mosquitoes were cold anesthetized and decapitated with the heads and bodies placed into separate 1.7 mL tubes (Eppendorf). A 400 µl aliquot of Dulbecco's minimal essential medium (DMEM) (Gibco) supplemented with 10% fetal bovine serum (FBS), 100 U/mL of penicillin and streptomycin, 1 U/mL of fungizone and gentamycin was added to each tube and the sample was homogenized using a micropestle (Kontes). The supernatant was clarified by filtration through a 0.2 µM syringe filter (Pall) and stored at −70°C until use [36].

Virus presence was determined using the virus isolation method as described above. An infected mosquito exhibited a virus positive body [percent infected  =  (number positive bodies/total number of mosquitoes processed) X 100] while those with disseminated infections were the infected individuals with virus in the head [percent disseminated  =  (number of positive heads/number of positive bodies) X 100]. Quantities of viral RNA were determined using real-time RT-PCR (above) and correlated with viral titer.

## Results

### Adult mosquito field collections

Adult mosquitoes were captured using three different collection methods (light trap, gravid trap, and vacuum aspirations). A total of 879 mosquitoes were collected in 84 trap nights. Additionally, 475 individuals collected as larvae and/or pupae were reared to adults for confirmatory identification and processing. Nine species were identified in these collections (**Table 2**). The most abundant adult species collected was *Aedes hensilli* (41.2%) followed by *Culex quinquefasciatus* (28.1%). All other species each comprised less than 10% of the total collection. All adult mosquitoes (field collected adults and those reared from immatures) were processed and subjected to virus isolation efforts. No viable virus was recovered from any of these mosquitoes.

**Table 2:** Summary of mosquito species collected as adults using three collection methods.

*Species*	*Collection Method(s)*	*% of total adult collection* (n)
*Aedes aegypti*	Aspiration	0.1 (1)
*Aedes hensilli*	Aspiration, gravid trap, light trap	41.2 (362)
*Aedes vexans*	Aspiration, gravid trap, light trap	1.6 (14)
*Coquillettidia crassipes*	Gravid trap, light trap	6.0 (52)
*Culex gossi*	Aspiration, gravid trap, light trap	8.0 (70)
*Culex nigropunctatus*	Aspiration, gravid trap, light trap	8.9 (78)
*Culex quinquefasciatus*	Aspiration, gravid trap, light trap	28.1 (247)
*Culex sitiens*	Aspiration, gravid trap, light trap	4.3 (38)
*Lutzia fuscana*	Light trap	0.5 (4)

### Immature mosquito field collections

**Figure 2:**
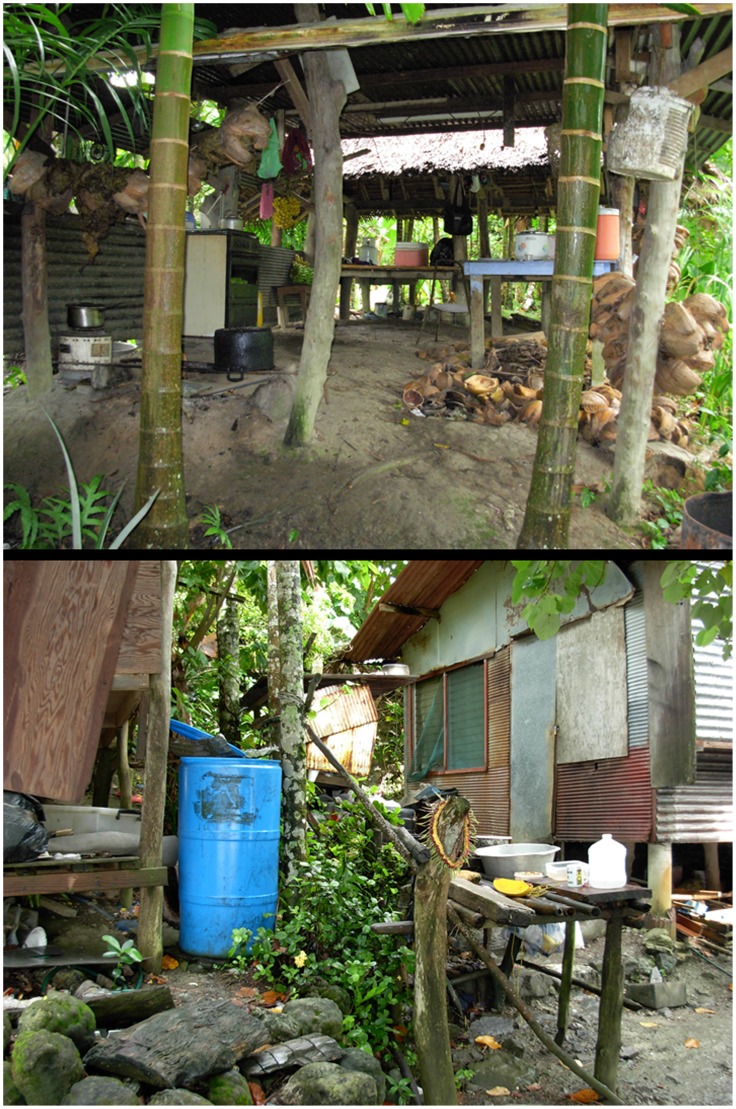
Typical water holding containers at individual homes including water barrels, coconut shells and cooking utensils.

From 170 randomly surveyed households (July 4–16, 2007), 1366 water holding habitats were identified. Larvae and/or pupae were collected from 586 of these containers and 85% of surveyed households had at least one infested habitat; individual habitats sometimes contained more than one species (**Figure 2**). The most prevalent containers with larvae or pupae were discarded cans followed by coconut shells (**Table 3**). Proportionally, containers including tires, tarps, floats, and bamboo had high percentages of immatures but several of these container types were found only infrequently (**Table 4**). Containers such as water barrels, used to collect rainwater, while proportionally fewer in number than other containers, were actually major contributors to mosquito production due to the sheer number of larvae and pupae present (e.g. thousands of immature mosquitoes per water barrel in comparison with cans or shells which typically contained fewer than 10 individuals each). In total, ten different species were identified from the larval collections. *Ae. hensilli* was both the most abundant and most prevalently identified immature species being found in 83% of the infested containers distributed all over the island (household index of 81.2 and Breteau index of 282.9).

**Table 3:** Total number of each container type infested with immature forms of each mosquito species.

Container	*Aedes aegypti*	*Aedes hensilli*	*Aedes lamelliferus*	*Aedes maehleri*	*Aedes vexans*	*Culex gossi*	*Culex nigropunctatus*	*Culex quinquefasciatus*	*Culex spp.*	*Lutzia fuscana*
Animal pan		1					1	1		
Bamboo							1	1		
Boat		2								
Bottle	2	31	1			1	1	1		
Bucket	5	55	2	2		3	5	3		2
Can(s)	2	113	4	3			5	4		1
Coconut shell(s)	1	90	7	2	1		6	4		
Cooking items	1	50	2	1		3	7	3		
Float		6		1			1			
Flower pot		11						1		
Ground pool	1	17			4	1	2	1	1	2
Live plant/axil		12	3	3	4		2	2		1
Plant frond	1	10					1			
Tarp	1	8	1				2			1
Tire(s)	5	48	1	11	1	2	1			7
Water barrel	7	30	2	1			3	5		1
Water tank		2						1		

**Table 4:** Proportion of water-holding containers infested with larvae and/or pupae.

	*Number containing larvae/pupae*	*Number without larvae/pupae*	*Proportion infested (%)*
Tire(s)	58	31	65
Tarp	11	7	61
Float	5	4	56
Coconut shell(s)	95	89	52
Bamboo	1	1	50
Can(s)	124	131	49
Bottle	32	35	48
Cooking items	57	65	47
Bucket	64	90	42
Water barrel	45	76	37
Flower pot	12	23	34
Live plant/axil	22	50	31
Boat	2	5	29
Plant frond	14	49	22
Ground pool	25	92	21
Water tank	2	8	20
Animal pan	2	10	17

### Laboratory infections

Because no virus was found in any of the field collected material, laboratory infections were performed on the most common mosquito collected, *Ae. hensilli*, to determine if this species could have served as the epidemic vector. Cohorts of *Ae. hensilli* were infected with three different viruses during these studies: 1) ZIKV - to determine if this was the likely vector during the outbreak; 2) CHIKV- to ascertain whether *Ae. hensilli* could serve as a vector for this virus which was expanding through SE Asia and was considered as a possible etiology of the outbreak prior to ZIKV diagnosis; 3) DENV - as *Ae. hensilli* was previously postulated as the vector of the 1996 dengue outbreak in Yap [5].

Cohorts of 3–4 day old adults were provided infectious blood meals with titers of at least 4.9 log_10_ pfu/mL. Mosquitoes provided the lowest dose of ZIKV were resistant to infection with only 7% becoming infected (**Table 5**). However, at least 80% of those receiving a slightly higher dose became infected. Curiously, only 13–23% of those developed disseminated infections. Only a small percentage of mosquitoes exposed to DENV-2 became infected (0–21%) and few of these had virus dissemination. In contrast, *Ae. hensilli* was found to be exceptionally sensitive to CHIKV with infection and dissemination rates greater than 60% and 80% respectively.

**Table 5:** Infectivity, dissemination, and viral tissue titers of *Aedes hensilli* mosquito heads and bodies on day 8 post infection.

Virus (Strain)	Rep.	Titer (log_10_ pfu/mL)	% infection (n)		Average titer in log_10_ pfu equivalents/mL (range)	
			Body	Head	Body	Head
Zika (MR766)	1	4.9	7.1 (14)	0 (1)	3.1	na
	2	5.7	80.0 (20)	12.5 (16)	2.7 (1.0–3.3)	1.8 (1.0–2.1)
	3	5.9	86.1 (36)	22.6 (31)	3.2 (1.0–4.0)	2.0 (0.6–2.7)
DENV-2 (TR1751)	1	5.3	20.7 (29)	16.7 (6)	2.6 (0.1–3.23)	2.1
DENV-2 (Jam1409)	1	5.5	0 (20)	0	0	na
CHIKV (COM 125)	1	5.7	62.5 (32)	80.0(20)	5.0 (1.0–5.6)	4.1 (1.1–4.4)

## Discussion

The 2007 outbreak of ZIKV in Yap prompted the investigation of vectorial capacity of the predominant local mosquito to transmit this virus and other related viruses that are present or threaten to affect FSM and other Western Pacific island countries. Yap State, the western-most part of FSM, has previously been affected by arboviral outbreaks [6] but the discovery of ZIKV on the island highlighted the risk of epidemics due to agents previously unknown to the area. During the entomological investigations, collection of mosquito larvae and pupae from over 15 distinct container types revealed a wide range of habitats, both natural and artificial, that could support development of a variety of mosquito species. Because the island extensively imports products via cargo ships, introduction of exotic species that could utilize the variety of habitats is a strong possibility. This could allow further novel arboviral introduction events on the island. For example, *Ae. albopictus* could easily be or have been introduced to the island due to the proximity and intense air and sea traffic with Guam and Mariana Islands where this species is widespread [37]. None were found during this study.

The overwhelmingly predominant mosquito species found on the island was *Ae. hensilli*. This mosquito was previously speculated to be the vector of DENV during the 1995 outbreak in Yap State as it was the only *Aedes (Stegomyia)* present on some affected islands [6]. However, like in this outbreak, no isolations were made from field-collected mosquitoes and no arboviruses have ever been reported from this species so incrimination as a vector could not be biologically confirmed. The collection of additional mosquitoes may have allowed virus isolation from field material but repeated strong rainstorms limited the number of adults collected. As in the previous dengue outbreak, *Ae. hensilli* is the most probable outbreak vector due to its high density, widespread distribution on the island, and its tendency to bite humans. Although transmission studies may have helped clarify vector status, laboratory infection studies reported here further suggest that this is a probable vector due to the high infection rates with ZIKV. While there is an admittedly suboptimal dissemination rate to indicate vector status for ZIKV, there has been documentation of other *Aedes (Stegomyia)* mosquitoes serving as outbreak vectors even with low susceptibility to infection or dissemination. For example, *Ae. aegypti*, which has been reported to be relatively resistant to infection to yellow fever virus, has nevertheless been implicated in outbreaks of yellow fever [38]. Vector status of *Ae. hensilli* for DENV-2 is more difficult to assert based on the laboratory data indicating less than 20% infection rates with virtually no dissemination. However, susceptibility to viruses in at least 2 distinct arboviral genera (*flavivirus* and *alphavirus*) suggests that this species could possibly serve as a vector of other medically important arboviruses typically transmitted by *Aedes (Stegomyia)* species (e.g. yellow fever and chikungunya viruses). It could also serve as a vector of arboviruses in large population centers where the mosquito is found [39]. *Aedes hensilli* has a limited known distribution consisting of FSM, Palau, and Singapore [39] suggesting that these additional areas might also be potentially at risk due to arboviral pathogens vectored by this species.

Since little is known of the biology or zoonotic transmission of ZIKV, it is also possible that other *Scutellaris* group species (among others) could be possible vectors of the virus. This is supported by the findings that ZIKV has previously been associated with *Ae. africanus* [23], [40], [41], *Ae. luteocephalus* [42], and *Ae. aegypti* [15] mosquitoes. There are numerous *Scutellaris* group mosquitoes from island ecologies including *Aedes cooki*, *Aedes polynesiensis*, *Aedes palauensis*, *Aedes rotumae*, and *Aedes scutellaris*, and others, some of which have been implicated in arboviral transmission [42]–[48]. The range of the *Scutellaris* group mosquitoes should be considered as possible vectors of ZIKV in islands of the Pacific and elsewhere.

*Aedes hensilli* was found to be very susceptible to infection by CHIKV. This finding was interesting as the strain of CHIKV selected was a Central/East African genotype strain associated with the Indian Ocean lineage but not possessing the valine residue at E1that has been linked to increased infectivity in *Ae. albopictus* [49]. A strain without this mutation was specifically selected to evaluate the susceptibility of *Ae. hensilli* to a virus that may not have been adapted to alternate *Scutellaris* group mosquitoes. However, the high degree of susceptibility to CHIKV even without the valine reside at position 226 is not completely unexpected as distinct populations of *Ae. albopictus* have historically shown significant susceptibility to CHIKV [50]. The ability of *Ae. hensilli* to be infected with CHIKV again, like with ZIKV, indicates that geographic areas with less well characterized *Scutellaris* group mosquitoes should consider alternate species to be potential vectors of introduced arboviral diseases.
